# Supplementary material for: Activated Carbon and Carbon Quantum Dots/Titanium Dioxide Composite Based on Waste Rice Noodles: Simultaneous Synthesis and Application in Water Pollution Control
Source: Nanomaterials (Basel). 2022 Jan 29;12(3):472. doi: 10.3390/nano12030472 (PMC8838941; doi:10.3390/nano12030472)
Supplement: Supplementary file 1 [file nanomaterials-12-00472-s001.zip › nanomaterials-1544524-supplementary.pdf]

nanomaterials

Supplementary Information

Activated Carbon and Carbon Quantum Dots/Titanium Dioxide Composite Based on Waste Rice Noodles: Simultaneous Synthesis and Application in Water Pollution Control

Xinyan Jin [1], Ruijie Che [1], Jie Yang [1], Yan Liu [1], Xinbao Chen [1],·Yunge Jiang [1], Jiaqi Liang [1], Shuoping Chen [1,*] and Heping Su [2,*]

[1] College of Materials Science and Engineering, Guilin University of technology, Guilin 541004, China; 2120200261@glut.edu.cn (X.J.); 2120210253@glut.edu.cn (R.C.); 2120180167@glut.edu.cn (J.Y.); 2120200279@glut.edu.cn (Y.L.); 3172042041419@glut.edu.cn (X.C.); 3192042041221@glut.edu.cn (Y.J.); 3192042041306@glut.edu.cn (J.L.)
[2] College of Science, Guilin University of Technology, Guilin 541004, China
* Correspondence: 2012014@glut.edu.cn (S.C.); 2009059@glut.edu.cn (H.S.)

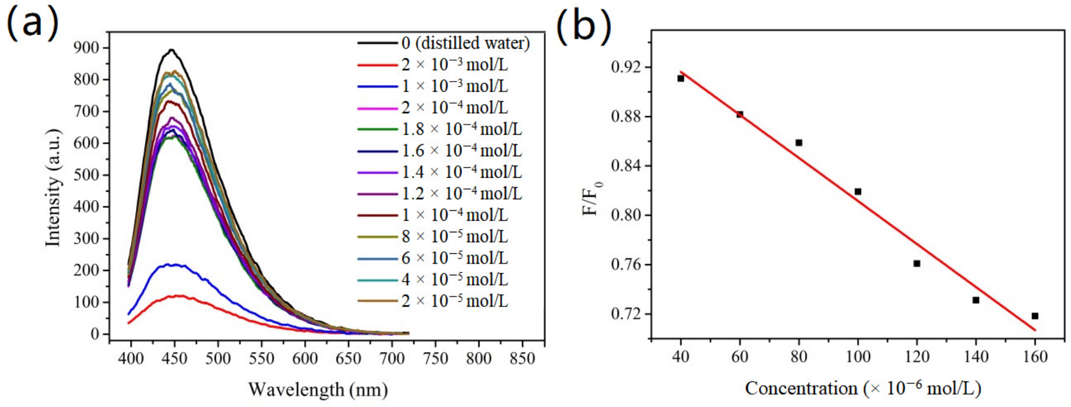

Figure S1. (**a**) Fluorescence response of different concentrations of Fe^{3+} solution to CQDs based on WRN; (**b**) Linear relationship between fluorescence response of CQDs and Fe^{3+} solution concentration.

The photoluminescence spectra and Fe^{3+} detection of CQDs were examined with a Varian Cary Eclipse fluorescence spectrophotometer (Palo Alto, CA, USA) with a xenon lamp as the excitation source in the range of 380 to 720 nm and an excitation wavelength of 340 nm. The CQDs showed blue fluorescence with an emission peak at 423 nm with a fluorescence rate 19.22%. In liquid samples containing Fe^{3+}, evidently fluorescence quenching was observed with CQDs and its fluorescence intensity decreased with the increasing of Fe^{3+} concentration. In Fe^{3+} solution with a concentration range from 4×10^{-5} mol/L~16×10^{-5} mol/L, the fluorescence response (F/F_0) of CQDs/PLA device was linearly correlated with Fe^{3+} concentration. After fitting, a linear equation could be obtained, in which $F/F_0 = 0.986 - 0.00174C_{Fe}$, with $R_2 = 0.97$, and the lowest limit of detection for quantitative analysis was calculated to be 3.34×10^{-5} mol/L.

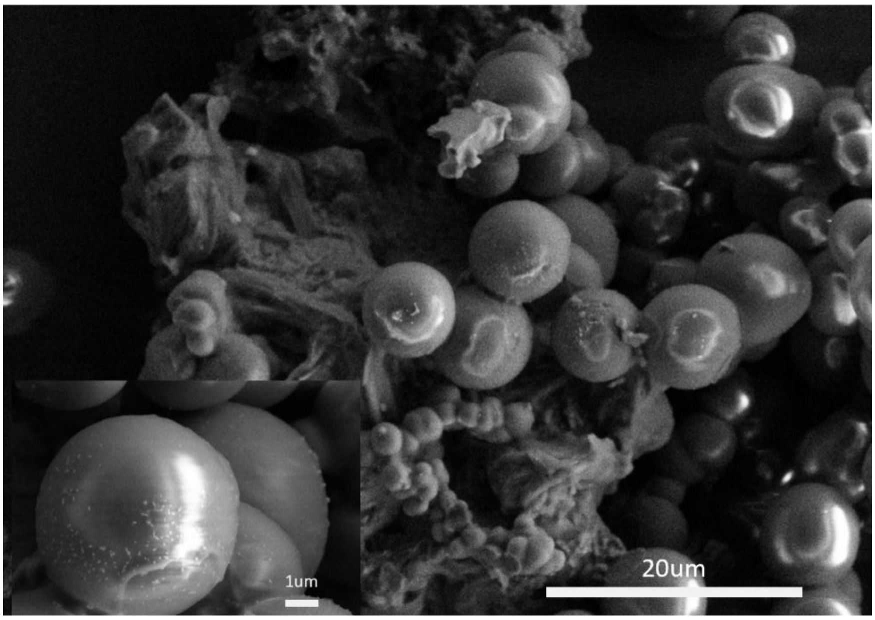

Figure S2. The SEM image of HTC.

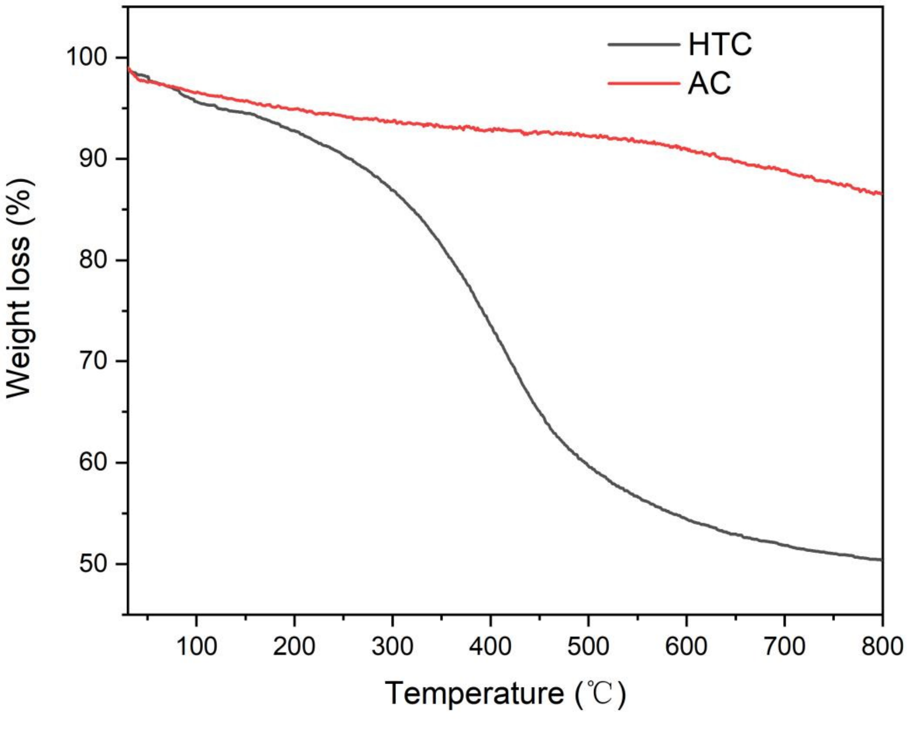

Figure S3. The TG curves of HTC and AC based on WRN.

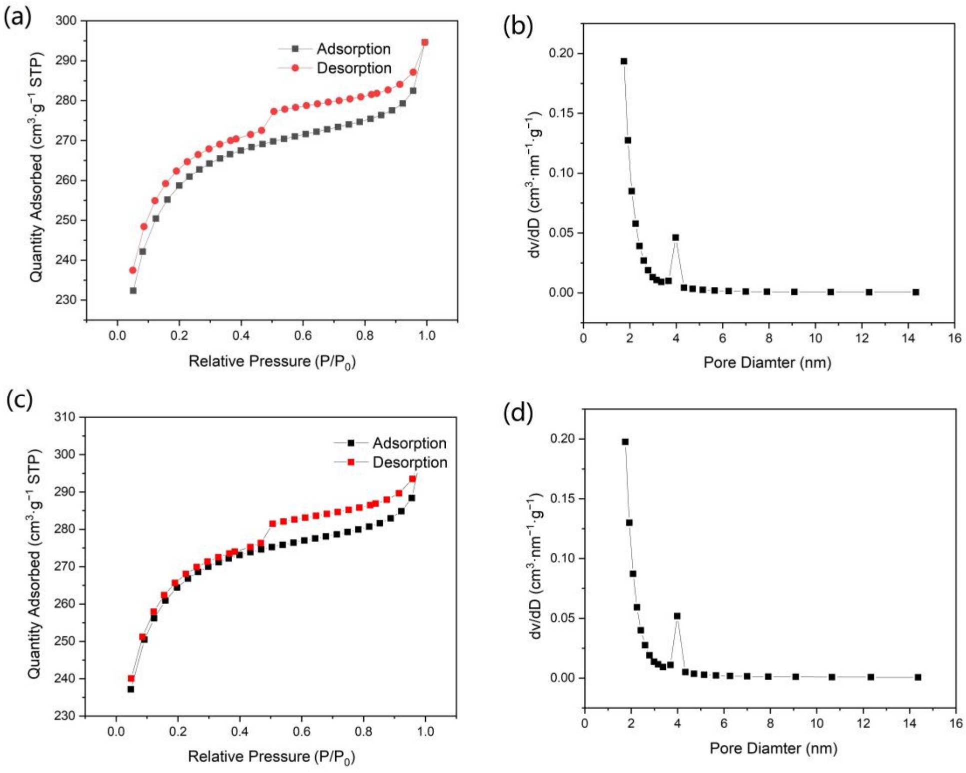

Figure S4. (**a**,**b**): The nitrogen adsorption/desorption isotherms (**a**) and pore size distribution curves (**b**) of AC after Pb(II) adsorption; (**c**,**d**): The nitrogen adsorption/desorption isotherms (**c**) and pore size distribution curves (**d**) of AC after Cr(VI) adsorption.

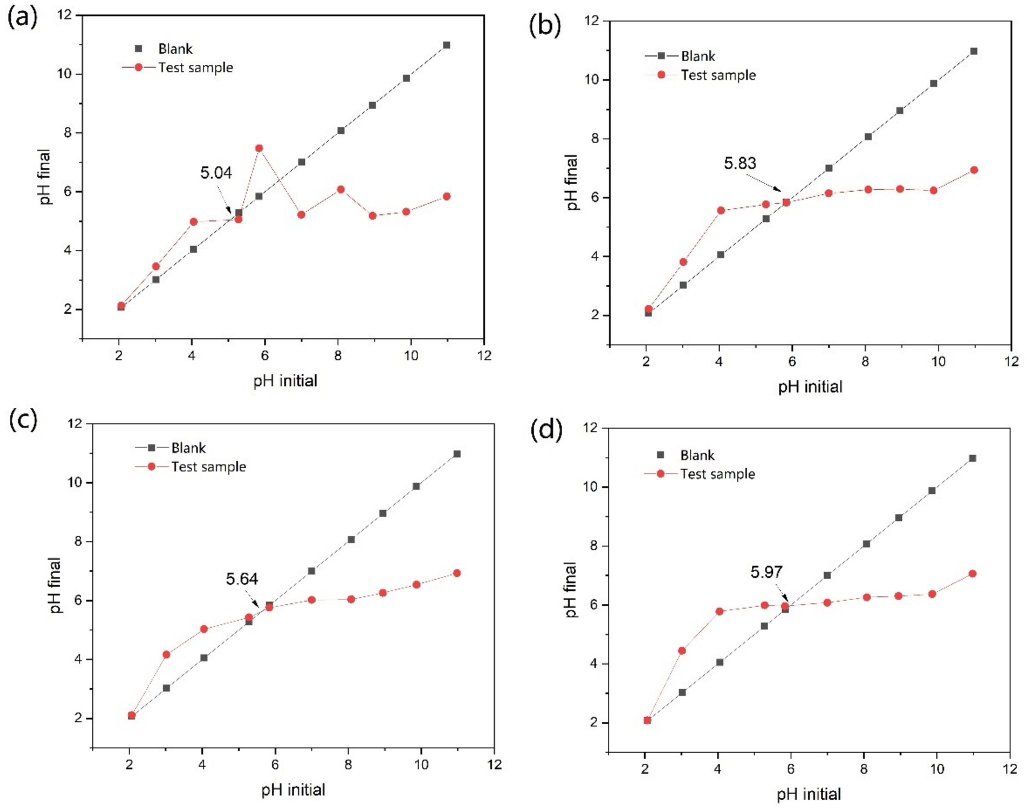

Figure S5. The PZC of HTC (**a**), AC (**b**), AC after Pb(II) adsorption (**c**) and AC after Cr(VI) adsorption (**d**).

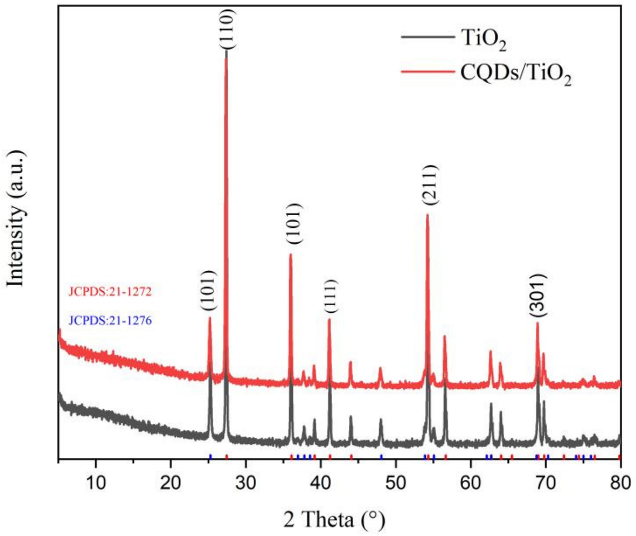

Figure S6. PXRD patterns of CQDs/TiO_2 composite and pure nano-TiO_2 powder.

The diffraction peaks at 25.28, 48.05, 53.89, 62.6°, 68.76 and 70.31° could be attributable to anatase TiO_2 (JCPDS card No. 21-1272), while the diffraction peaks at 27.44, 36.09, 41.23, 54.32 and 69.01° indicated the existence of rutile TiO_2 (JCPDS card No. 21-1276).

Thus, the TiO_2 in the resulted composite was a mixture of anatase and rutile. Due to low content, the diffraction peaks of CQDs in the composite could not be observed obviously.

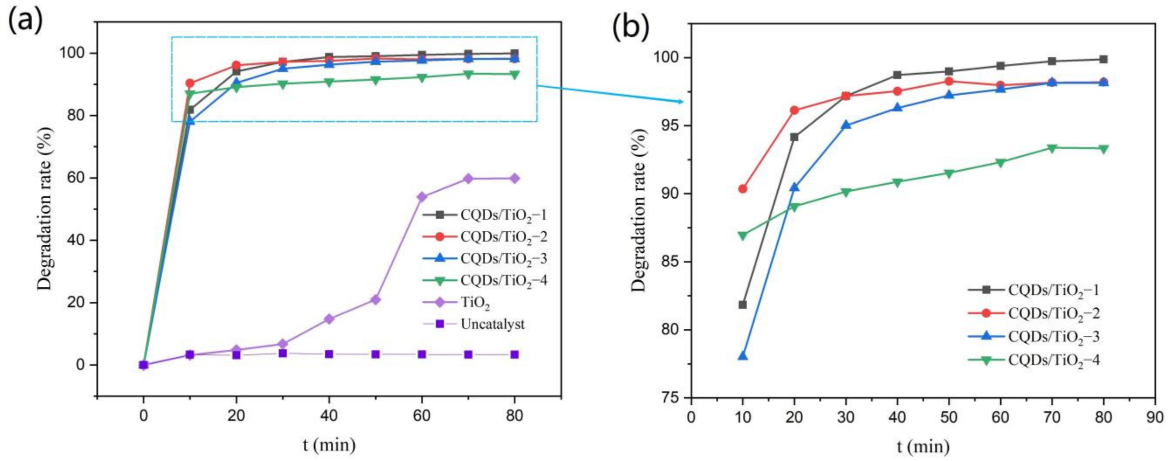

Figure S7. (**a**) The photocatalytic degradation rate of CQDs/TiO_2 composites with different carbon contents and pure TiO_2 to methylene blue within different irradiation times under 405 nm purple light; (**b**) The partial enlargement of (**a**) showing the degradation rates of CQDs/TiO_2 composites with different carbon content to methylene blue.

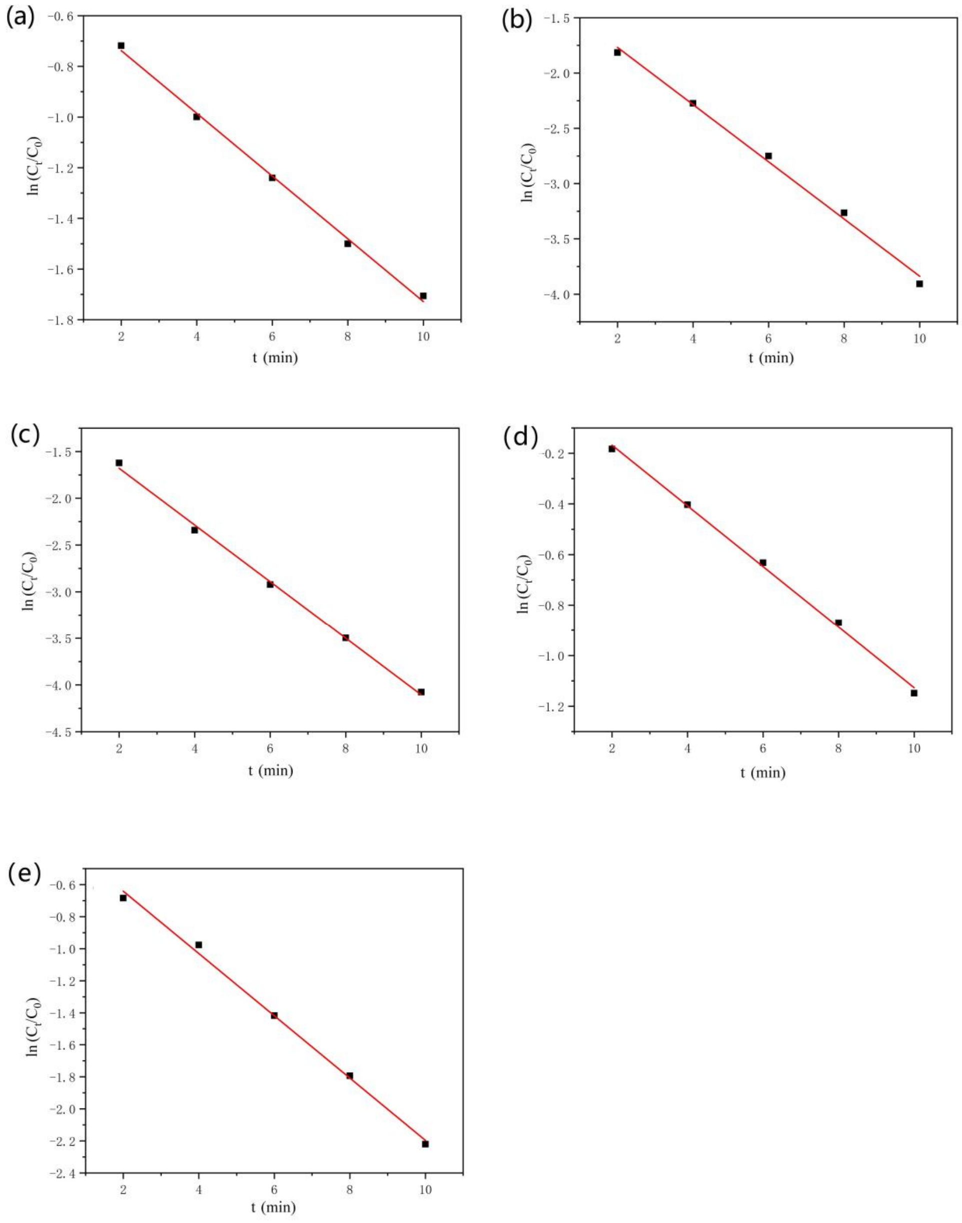

Figure S8. The kinetic fitting of photocatalytic degradation of CQDs/TiO_2 composite to methylene blue (**a**), malachite green (**b**), methyl violet (**c**), basic fuchsin (**d**) and rhodamine B (**e**) under 405 nm purple light.

Table S1. The optimization of the experimental conditions of AC.

Serial Number	Mass Ratio of HTC Powder to KOH	Activation Temperature (°C)	Activation Time (min)	Surface Area (m^2/g)
A	5:1	800	60	122.72
B	1:1	800	60	737.48
C	1:2	800	60	603.12
D	1:3	800	60	547.62
E	1:2	700	60	615.07
F	1:2	750	60	367.54

G	1:2	850	60	644.47
H	1:2	800	30	958.2565
I	1:2	800	90	1250.35
J	1:2	800	120	1234.16

The optimization experimental conditions of AC (sample I) are as follows: Dosage of KOH (activation regent): twice as the mass of hydrothermal carbon; Activation temperature: 800 °C; Activation time: 90 min.

Table S2. Experimental conditions of CQDs/TiO_2 composite based on WRN.

Serial Number	Dosage of CQDs Solution (mL)	Dosage of Nano-TiO_2 Powder (g)	Carbon Content (wt%)
CQDs/TiO_2-1	200	2.5	15.24
CQDs/TiO_2-2	200	5	8.39
CQDs/TiO_2-3	200	7.5	5.91
CQDs/TiO_2-4	200	10	4.96
